# Polymer Dielectric-Based Emerging Devices: Advancements in Memory, Field-Effect Transistor, and Nanogenerator Technologies

**DOI:** 10.3390/mi15091115

**Published:** 2024-08-31

**Authors:** Wangmyung Choi, Junhwan Choi, Yongbin Han, Hocheon Yoo, Hong-Joon Yoon

**Affiliations:** 1 Department of Semiconductor Engineering, Gachon University, Seongnam 13120, Republic of Korea; dhkdaud217@gachon.ac.kr (W.C.); gksdydqls125@gachon.ac.kr (Y.H.); 2 Department of Chemical Engineering, Dankook University, Yongin 16890, Republic of Korea; jhchoi2301@dankook.ac.kr; 3 Department of Electronic Engineering, Gachon University, Seongnam 13120, Republic of Korea

**Keywords:** polymer dielectric, memory devices, field-effect transistors, triboelectric nanogenerators, flexible electronics, self-powered systems

## Abstract

Polymer dielectric materials have recently attracted attention for their versatile applications in emerging electronic devices such as memory, field-effect transistors (FETs), and triboelectric nanogenerators (TENGs). This review highlights the advances in polymer dielectric materials and their integration into these devices, emphasizing their unique electrical, mechanical, and thermal properties that enable high performance and flexibility. By exploring their roles in self-sustaining technologies (e.g., artificial intelligence (AI) and Internet of Everything (IoE)), this review emphasizes the importance of polymer dielectric materials in enabling low-power, flexible, and sustainable electronic devices. The discussion covers design strategies to improve the dielectric constant, charge trapping, and overall device stability. Specific challenges, such as optimizing electrical properties, ensuring process scalability, and enhancing environmental stability, are also addressed. In addition, the review explores the synergistic integration of memory devices, FETs, and TENGs, focusing on their potential in flexible and wearable electronics, self-powered systems, and sustainable technologies. This review provides a comprehensive overview of the current state and prospects of polymer dielectric-based devices in advanced electronic applications by examining recent research breakthroughs and identifying future opportunities.

## 1. Introduction

Polymer dielectric materials have become increasingly important in electronic devices because of their unique electrical, mechanical, and thermal properties [1,2,3,4]. Unlike traditional inorganic dielectrics, polymers offer flexibility, lightweight characteristics, and ease of processing, making them ideal for various applications. The ability to modify the chemical structure of polymers allows for the adjustment of their dielectric properties, enabling the design of materials with high dielectric constants, low dielectric loss, and excellent insulating performance [5,6,7,8]. These attributes are critical for developing high-performance electronic devices that meet the demands of modern technology.

The versatility of polymer dielectric materials has led to their application in various emerging electronic devices, including memory devices [9,10,11], field-effect transistors (FETs) [12,13,14,15], and triboelectric nanogenerators (TENGs) [16,17,18]. In memory devices, polymer dielectrics can improve crystallinity through molecular structure design, thereby providing stable switching characteristics in memristors [19]. Additionally, polymer dielectrics have the ability to trap electrostatic charges, resulting in a quasi-permanent electric field, making them useful as charge-trapping layers [20]. By controlling the composition of the material, the characteristics of the device can be adjusted [21,22]. For FETs, polymer dielectrics induce the field-effect when voltage is applied to a gate electrode to accumulate charge carriers while blocking leakage current. Also, polymer dielectric layers provide the interface with semiconducting layers, significantly affecting charge carrier movement in the channel. Thus, polymers in FETs determine the power consumption, operational stability, and reliability of the devices [23,24,25]. TENGs, which convert mechanical energy into electrical energy, benefit from the high charge-trapping and transfer capabilities of polymer dielectrics [26,27], leading to improved energy-harvesting efficiency. Polymer dielectrics in TENGs improve electrical output by increasing the charge generation and storage capacity, primarily due to their high dielectric constant and effective surface properties. This leads to higher surface charge density, which directly relates to improved performance [26]. Additionally, their mechanical flexibility and durability allow TENGs to maintain consistent performance over cycles, making them ideal for applications that require repeated mechanical stress. The integration of these devices into flexible and wearable electronics highlights the importance of polymer dielectrics in advancing next-generation technologies [28,29].

Polymer dielectrics have superior electrical and mechanical properties to other dielectric materials, making them a material of choice for numerous applications [30,31,32,33]. Their high dielectric strength and processability allow for the fabrication of devices with complex geometries and large surface areas. Through appropriate molecular design, polymer dielectric materials are able to exhibit intrinsic flexibility and stretchability, making them key components in flexible and stretchable devices [34,35,36,37,38,39,40]. Their high dielectric strength, combined with excellent flexibility and processability, allows for the fabrication of devices with complex geometries and large surface areas. In addition, polymers can be processed using various methods, including solution casting [41,42,43], spin coating [44,45,46,47], and printing [47,48,49], facilitating large-scale manufacturing. These properties enhance the performance and reliability of electronic devices and open new possibilities for innovative applications in flexible, stretchable, and wearable electronics.

This paper reviews the recent advances in polymer dielectric materials and discusses their applications in memory devices, FETs, and TENGs. The focus is on the unique properties that make these materials suitable for these applications, the challenges faced in their implementation, and the potential future developments that can further enhance their performance. The paper provides a comprehensive overview of their role in advancing modern electronic technologies by examining the synergies and integration possibilities of these polymer-based devices.

## 2. Polymer Dielectric-Based Electronics

### 2.1. Memory Devices

Memory is an electronic device that stores information through changes in the programming and erasing states. They can provide different output currents up to several orders of magnitude depending on the programming (*V*_prog_) and erasing voltages (*V*_eras_) [44,50]. Therefore, the performance of a memory device is determined by the difference in the current–voltage characteristics between the programming and erasing states, known as the memory window, and the number of states that can be produced within this range. In addition, a fast memory speed is generally desirable for high-speed computing, which is determined by the extent of the memory window that can be achieved based on the duration for which *V*_prog_ and *V*_eras_ are applied. Memory devices can be categorized as volatile or non-volatile based on the charge retention capability of the stored data. Another important characteristic for evaluating memory devices is endurance [51]. This metric assesses whether the memory can be operated reliably in response to repeated programming and erasing operations.

Memory devices based on polymer dielectrics have been developed where the key performance indicators for memory devices have been investigated, including low operating voltage, long retention time, and stable cycle-to-cycle endurance, even after multiple cycles [52,53]. These characteristics can vary according to the specific memory structures and material combinations. In particular, using polymer insulators allows for inherent mechanical flexibility, enabling the development of flexible or stretchable memory devices [54,55]. This paper revisits the latest results on memory devices using polymer dielectric materials as active layers. The representative memory performance and their structures and combinations are examined.

### 2.2. Field-Effect Transistors (FETs)

A FET is an essential component in modern electronics, acting as a switch or amplifier by differentiating the on- and off-states. Hence, increasing the on-current while maintaining the reduced off-current is crucial to maximizing their functions. The gate dielectric layer plays a vital role in FET operation, which blocks unwanted leakage current between the gate and source/drain electrodes in the off-state, accumulating charge carriers at the interface between the gate dielectric layer and the semiconductor in the on-state. In the FET operation, the drain current (*I*_D_) can be expressed using the following equations:

(1) $$I_{D,\mathrm{lin}}=\left(\frac{W}{L}\right)\mu_{\mathrm{lin}}C_{i}\left(V_{G}-V_{T}-\frac{V_{D}}{2}\right)V_{D}$$

(2) $$I_{D,\mathrm{sat}}=\left(\frac{W}{2L}\right)\mu_{\mathrm{sat}}C_{i}{\left(V_{G}-V_{T}\right)}^{2}$$

where *I*_D,lin_ and *I*_D,sat_ are the drain currents in the linear and saturation regime, respectively, and *W* and *L* are channel width and length, respectively. *μ*_lin_ and *μ*_sat_ are the linear and saturation mobility, respectively, and *C*_i_ is the capacitance per unit area of the dielectric layer. *V*_G_, *V*_T_, and *V*_D_ are the gate voltage, threshold voltage, and drain voltage, respectively. The current output of FETs is proportional to the *C*_i_ value of the gate dielectric layer, which is determined using the following equation:

(3) $$C_{i}=\varepsilon_{0}\frac{k}{d}$$

where *ε*_0_, *k*, and *d* are the vacuum permittivity, dielectric constant, and thickness of the gate dielectric layer, respectively. Therefore, it is highly desirable to increase the dielectric constant and decrease the thickness of the dielectric layer to maximize *I*_D_ under low operating voltages. An appropriate dielectric strength and low leakage current should be secured for the reliable operation of FETs. Exploiting advantages, such as cost-effectiveness, mechanical deformability, and tunability in their chemistry, polymer dielectric materials have been used widely to achieve reliable operation and optimize the electrical characteristics of emerging FET devices [56,57,58,59].

### 2.3. Triboelectric Nanogenerators (TENGs)

Since the research on TENGs was first reported in 2012 [16], the research field of TENGs has been highlighted as one of the promising energy-harvesting technologies. Triboelectricity is generated by contact charging and electrostatic induction between triboelectric materials with different dielectric properties. The dielectric constant is the central parameter that describes how efficiently the active material is polarized when subjected to an electric field and is calculated using Equation (4) [60]:

(4) $$k=\frac{C\times d}{\varepsilon_{0}\times A}$$

where *k*, *C*, *d*, and *A* are the dielectric constant, capacitance, thickness of the active layer, and area of the active layer, respectively. In the metal−insulator system, the surface charge transfer between the two layers can be induced as follows:

(5) σ=W−E0e1/kt+1zε0+1/NsE¯e2

where *σ*, *W*, *E*_0_, *t*, *z*, *e*, and $\bar{N_{s}\left(E\right)}$ represent the surface charge density on the dielectric material, the metal’s work function, the effective work function, the thickness of the insulator, the gap between the metal and the insulator layer, the elementary charge, and the average surface density of states in the dielectric layer, respectively [61]. Equation (5) suggests that a thinner dielectric layer combined with a larger ε/t ratio can lead to an increased surface charge density. Consequently, the permittivity of the bulk insulator may play the most crucial role in influencing contact electrification among the various factors affecting surface charge density. The permittivity of the bulk insulator can be adjusted by incorporating a high-k material layer or by embedding high-k particles. Additionally, high-k materials can act as charge-trapping layers, enhancing surface charge retention. This section explores strategies to enhance contact electrification by modifying the insulator’s permittivity.

## 3. Recent Advances in Electronic Devices

### 3.1. Memory Devices

#### 3.1.1. Polymer Dielectric-Based Memory Devices

Memory devices based on polymer dielectrics are being developed. The use of polymer insulators provides inherent mechanical flexibility, enabling the formation of flexible or stretchable memory devices [54,55]. The most frequently attempted memory devices based on polymer insulators are two-terminal memristor structures with a sandwich configuration [62,63], where the polymer insulator is placed in the middle. A memristor operates on the memory principle where the polymer insulator typically contributes by preventing a leakage current between the two electrodes, resulting in a high resistance state (HRS). On the other hand, when metal filaments form, the current increases sharply, leading to a low resistance state (LRS). The charge trap device is another structure for memory devices based on polymer dielectric materials [20,64]. An appropriate design of polymer dielectric can enhance the charge trap memory devices, where the charge trapping in a floating gate is facilitated within low *V*_prog_ and *V*_eras_. Moreover, trapping charges within the insulator to adjust the *V*_T_ of the transistor or using a polymer dielectric that induces surface charge traps as a storage layer enables charge trap-based memory operation without the need for a floating gate. This section divides the discussion into memristor-based and charge trap-based polymer memory devices. The focus is on highlighting recent developments and exploring the polymer materials, the device structures incorporating them, and the resulting memory device characteristics.

#### 3.1.2. Application of Polymer Dielectric Materials for Conductive Filament Memristors

**Figure 1:**
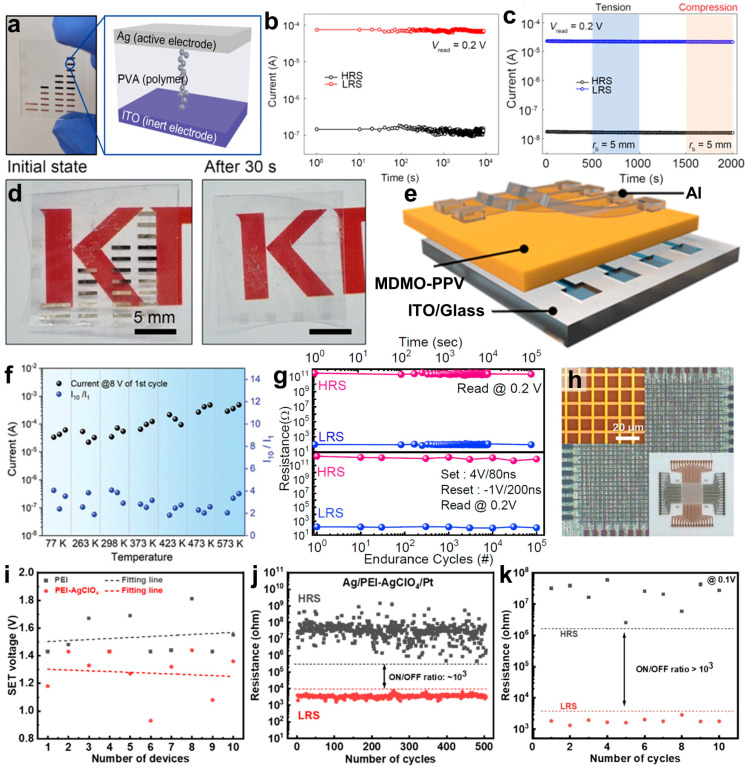
(**a**) Schematic diagram and photograph of PVA-based memristors. The memory retention characteristics of memristors investigated for (**b**) rigid substrate-based and (**c**) flexible substrate-based devices. (**d**) The photograph of the biodegradation process of a PVA-based memristor device at 300 K [71]. Copyright © 2023, John Wiley and Sons. (**e**) The schematic diagram of a memristor device based on MDMO-PPV, and (**f**) its memory retention characteristics at temperatures ranging from 77 to 573 K [79]. Copyright © 2023, John Wiley and Sons. (**g**) The memory retention characteristics of a pV3D3-based memristor device over time and endurance cycles, and (**h**) optical microscopy image of a 32 × 32 array of memristors [51]. Copyright © 2024, John Wiley and Sons. (**i**) Variation in the SET voltage with the presence of metal salt (AgClO_4_) in PEI-based memristors. (**j**) The memory retention characteristics of PEI-AgClO_4_-based memristor. (**k**) The resistance switching curve of PEI-AgClO_4_-based memristor after 30 days of exposure to air [83]. Copyright © 2022, American Chemical Society.

Polymer insulator-based memristors have attracted attention because of the low cost, simple processing, and flexibility of polymers [65,66,67,68]. As environmental concerns are increasing, biodegradable materials are also in demand [69,70]. For example, Oh et al. used poly(vinyl alcohol) (PVA) to develop an eco-friendly and flexible memristor (Figure 1a) [71]. As the molecular weight of PVA decreases, the memristor performance is improved because it allows for the increased free volume necessary for ion migration and metallization [72]. The optimized PVA-based memristor device remained stable for 10^4^ s without current degradation (Figure 1b). A device was fabricated on an ITO-coated polyethylene naphthalate (PEN) substrate using the excellent mechanical flexibility of PVA. The device showed stable operation under tensile and compressive stresses with a bending radius of 5 mm (Figure 1c). The high solubility of PVA in water enables the development of biodegradable electronic devices, as shown in Figure 1d [73,74]. The memristor device dissolves rapidly in distilled water at room temperature (within 30 s). These results suggest the potential of facilitating flexible electronic device production and addressing the issue of electronic waste [75].

Many reported memristors based on polymer materials often show poor thermal tolerance, which can degrade the device’s performance [76]. Poly(triphenylamine) (PTPA) and PVA have been reported to form robust polymer-based memristors because of their thermal stability [77,78]. Li et al. implemented a memristor using poly[2-methoxy-5-(3′,7′-dimethyloctyloxy)-1,4-phenylenevinylene] (MDMO-PPV), showing robust performance in both extremely low- and high-temperature environments [79]. The MDMO-PPV thin film was deposited on an ITO-patterned glass substrate using the spin-coating technique (Figure 1e), achieving more than 95% yield with consistent performance across an 8 × 8 array. The MDMO-PPV-based memristor devices demonstrated real-time measurements across an ultra-wide temperature range (173–473 K), exhibiting reliable operation from 77 to 573 K in cryogenic and high-temperature environments (Figure 1f). The memristor characteristics remained stable even at temperatures as low as 77 K for 72 h.

One notable issue in solution-processed polymer memristors is performance degradation due to impurities or residues [80]. This can be resolved using a solvent-free initiated chemical vapor deposition (iCVD) technique. Kim et al. reported memristor devices based on poly(1,3,5-trivinyl-1,3,5-trimethyl cyclotrisiloxane) (pV3D3) using iCVD, showing excellent stability [51]. pV3D3 exhibits high thermal and chemical stability because of its high crosslinking density resulting from three crosslinking sites [80,81]. These characteristics can enhance the durability of the device. Indeed, the memristor devices based on pV3D3 maintained robust electrical properties after 10^5^ s and 10^5^ endurance cycles (Figure 1g). They also exhibited reliable retention characteristics, even at 85 °C. Crossbar-array memristor devices fabricated in a 32 × 32 matrix were manufactured using the solvent-free iCVD technique (Figure 1h). This method ensures that all electronic devices operate properly without impurities or residues from solution processes.

Studies have attempted to improve the performance of polymer-based memristor devices by exploring the introduction of interfacial load layers containing active metal clusters and constructing active layers with oriented molecular chain arrangements to direct filament growth [67,82]. Zhang et al. enhanced the performance of memristors by adding the metal salt AlClO_4_ to polyethylenimine (PEI) [83]. Compared to pure PEI-based memristors, devices containing AgClO_4_ allowed for a large number of silver (Ag) ions in the PEI layer to move more quickly to the platinum (Pt) electrode, contributing to the formation of Ag filaments. This phenomenon reduced the operating voltage of the device and enhanced its energy consumption efficiency (Figure 1i). Furthermore, adding AgClO_4_ improved the non-volatile performance of the memristor (Figure 1j), and its switching characteristics remained unchanged, even after exposure to air for 30 days (Figure 1k).

In this chapter, we revisited the deposition methods according to the polymer dielectric-based material classification. Table 1 represents a detailed summary of the polymer dielectric-based memristor devices in terms of polymer dielectric materials, deposition methods, device performance, environmental stability, and biodegradability parameters.

#### 3.1.3. Application of Polymer Dielectric Materials for Charge Trap Memory Transistors

**Figure 2:**
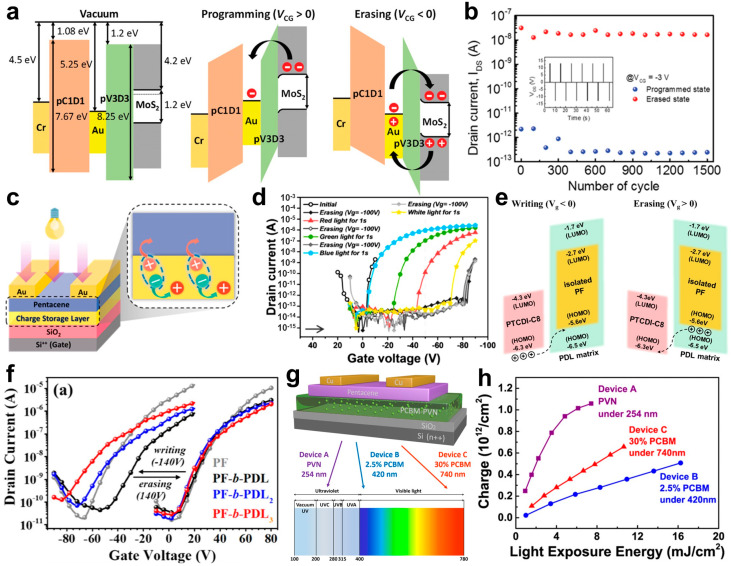
(**a**) Operating mechanism of MoS2-based memory for programming and erasing operations, and (**b**) memory retention characteristics under ±13 V with a pulse width of three seconds for operating gate pulses [50]. Copyright © 2019, John Wiley and Sons. (**c**) The schematic diagram of the photo-induced writing mechanism in pre-erased charge-trap polymer layer-based memory devices. (**d**) The writing/erasing characteristics of PFO charge-trap layer-based memory devices under various wavelengths [88]. Copyright © 2021, American Chemical Society. (**e**) The operating mechanism of PF-b-PDL-based memory for programming and erasing operations, and (**f**) the writing/erasing characteristics of memory devices based on the number of PDL arms in the PF-b-PDL structure [21]. Copyright © 2021, American Chemical Society. (**g**) The schematic diagram of the structure of PCBM/PVN blend-based memory devices and the selective photoresponse of the device according to the PCBM blending ratio. (**h**) The surface density of trapped charge versus light exposure energy per unit area curves of PCBM/PVN blend-based memory devices with different PCBM blending ratios [22]. Copyright © 2019, American Chemical Society.

Appropriate dielectric materials and thin thicknesses are required for floating gate-based transistor devices with a low operating voltage and stable endurance. The insulating layer preventing a leakage current at the control gate should be thin with a high dielectric constant to ensure low operating voltage. In contrast, the tunneling dielectric layer between the floating gate and the channel should use materials with a low dielectric constant to maintain charge trapping. Yang et al. developed MoS_2_-based low-voltage non-volatile memory devices using a high-*k* poly(2-cyanoethyl acrylate-co-diethylene glycol divinyl ether) [p(CEA-co-DEGDVE)] copolymer (pC1D1, *k*~6.2) that was synthesized from 2-cyanoethyl acrylate (CEA) and di(ethylene glycol) divinyl ether (DEGDVE), and a low dielectric constant pV3D3 polymer (*k*~2.2) [50]. The high energy barrier between MoS_2_ and pV3D3 enabled programming and erasing through tunneling, while gold nanoparticles (Au NPs) captured the charges from the deep quantum well (Figure 2a). The shallow lowest unoccupied molecular orbital (LUMO) level of pC1D1 prevented charges from moving to the control gate. In this energy configuration, the thin thickness and high dielectric constant of pC1D1 resulted in a low operating voltage (±13 V) and robust memory operation (Figure 2b). The low dielectric constant of pV3D3 helps prevent the de-trapping of the charges captured by the Au NPs, maintaining good performance (<10^5^ s).

A polymer electret is a dielectric material that can be used as a charge-trapping layer without a floating gate [87]. Chen et al. examined the characteristics of non-volatile memory transistors using poly(9,9-dioctylfluorene) (PFO) as the charge-trapping layer [88]. Using PFO as a charge-trapping layer in memory transistors resulted in semiconductor holes being trapped in the PFO layer during the electrical erasing process, shifting the *V*_T_ of the memory transistor in the direction of negative gate voltage (erasing). When PFO was exposed to light that is capable of activating it, PFO generated photo-generated electron–hole pairs. During this process, the photo-generated electrons recombine with the holes trapped in the PFO, neutralizing them, while the photo-generated holes transport to the organic semiconductor layer, maintaining the initial state (Figure 2c,d).

Additional materials are often added to polymer dielectrics [89,90] or block polymers [91,92], where two or more polymers are regularly linked within a single polymer chain to enhance the performance of memory transistors. This approach exhibits an operational mechanism similar to floating gate-based memory transistors. For example, Hsu et al. introduced a block copolymer of poly(9,9-di-n-hexyl-2,7-fluorene) and poly(δ-decanolactone) (PF-b-PDL) as the charge-trapping layer in memory transistors [21]. The PF chain served to trap holes, while the PDL chain acted as an insulating polymer serving as the dielectric layer and maintained the trapped charges in PF (Figure 2e). In the PF-b-PDL structure, as the PDL arm increased, the memory window increased from 63 V to 102 V (Figure 1f), and the memory retention characteristics were improved, maintaining a memory ratio of 3.5 × 10^4^. These attributes stem from the increased branching of PDL stabilizing the charges stored in PF. Zhang et al. developed memory transistors using phenyl-C_61_-butyric acid methyl ester (PCBM) and poly(2-vinyl naphthalene) (PVN), with the ability to detect and store light selectively based on the PCBM blending content (Figure 2g) [22]. The roles of PVN and PCBM in the device varied according to the blending content of PCBM. In a pure PVN device (Device A), the electrons generated by exposure to ultraviolet light were trapped in the PVN, enabling memory operation. In the device with 2.5% PCBM (Device B), when exposed to blue light, the electrons photoexcited in the PCBM were transported to and trapped in the PVN, with PCBM playing a supporting role. In the device with 30% PCBM (Device C), the electrons generated by red light were trapped in the PCBM, which functioned as a charge-trapping layer, while the PVN prevented electron detrapping from the PCBM. Each device demonstrated a correlation between the light exposure energy and the surface density of the trapping layer through variations in *V*_T_ with the exposure time. All the devices showed an increase in the surface density of the trapping layer as the light exposure energy increased, as shown in Figure 2h. The calculated sensitivity values were 2.3 × 10^11^ (Device A), 0.3 × 10^11^ (Device B), and 0.6 × 10^11^ mJ^−1^ (Device C).

To clearly present the characteristics of charge trap-based memory devices according to the types of polymer dielectrics and device types, we provided a detailed summary focusing on the structure and type of charge traps, as well as the device performance parameters (Table 2).

### 3.2. Field-Effect Transistors (FETs)

#### 3.2.1. Overview of Polymer Dielectric Materials for FET Devices

Various polymer gate dielectric materials have been studied extensively to accommodate the important requirements in FETs, including high carrier mobility (*μ*), low operating voltage, and device stability. High-*k* polymer dielectric materials have been designed to improve *C*_i_ by enhancing the polarization of the polymer materials [36,97]. Polyvinylidene fluoride (PVDF)-based ferroelectric polymers are one of the representative high-*k* polymer dielectric materials, which can also exhibit unique electrical properties, including negative capacitance (NC) behavior [98,99]. Reducing the thickness of the gate dielectric layer is another efficient way to increase the *C*_i_ value, and crosslinking has been explored to maintain the proper dielectric strength while decreasing the thickness of the dielectric layer [100]. Crosslinking is also important in improving mechanical deformability, which allows for designing stretchable polymer dielectric materials [101,102]. In addition to the gate dielectric layer, polymer dielectric materials have been introduced as doping and barrier layers to improve the charge transport characteristics and environmental stability, respectively [103,104].

Polymer dielectric materials have also been used to develop organic–inorganic hybrid dielectric layers, allowing for the synergistic effects of both organic and inorganic materials [105]. In layered hybrid dielectrics, the polymer dielectric layer can effectively passivate the surface trap sites of the inorganic dielectric layers and improve the mechanical flexibility of FET devices [106,107]. Furthermore, hybrid dielectric materials based on organic–inorganic mixtures have been developed, where inorganic nanoparticles or inorganic segments are incorporated in polymer matrices [108,109]. The following section highlights the recent research achievements in polymer-based dielectric materials for FET applications.

#### 3.2.2. Polymer-Based Dielectric Materials for FET Devices

**Figure 3:**
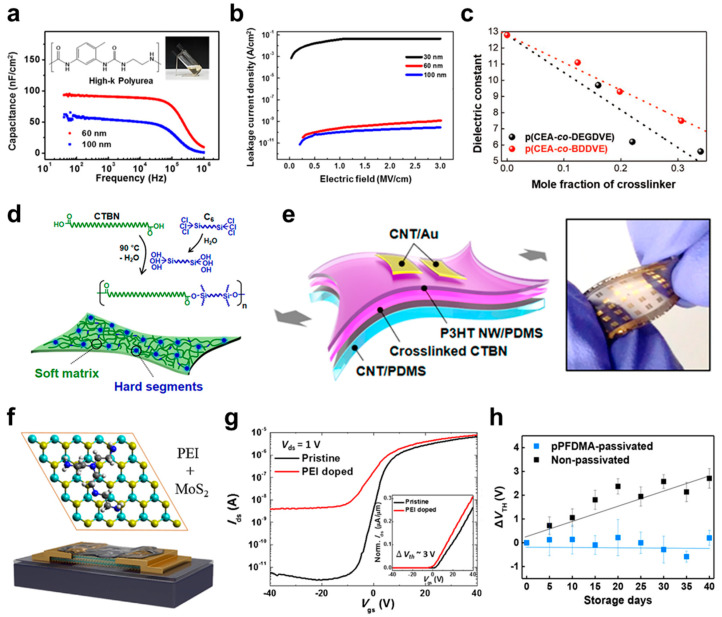
(**a**) *C*_i_−frequency (*f*) and (**b**) *J*−electric field (*E*) curves of polyurea with different thickness [97]. Copyright © 2018, American Chemical Society. (**c**) The change in dielectric constant according to the mole fraction of the crosslinker of p(CEA-co-DEGDVE) and p(CEA-co-BDDVE) [5]. Copyright © 2020, John Wiley and Sons. (**d**) The schematic diagram and the chemical structure of stretchable polymer dielectric. (**e**) The schematic diagram and photograph of P3HT NWs/PDMS-based stretchable organic FETs [101]. Copyright © 2018, American Chemical Society. (**f**) The schematic diagram of PEI-doped 2D MoS_2_ FET and (**g**) its transfer curve compared to the pristine device [150]. Copyright © 2017, John Wiley and Sons. (**h**) The change in *V*_T_ according to the storage time of pPFDMA-passivated and non-passivated nanoporous 2D MoS_2_ FETs [104]. Copyright © 2022, Springer Nature.

Polymer dielectric materials containing polar functionalities, such as cyano (−C≡N), hydroxyl (−OH), and epoxy groups, have been used to improve the dielectric constants [23,97,110,111,112,113]. For example, Yoo et al. developed high-*k* polyurea gate dielectric materials where the polar urea bonds were responsible for a high dielectric constant [94]. Polyurea dielectric materials were synthesized from different diisocyanate and diamine monomers. The effects of hydrogen bonding on dielectric properties were investigated systematically by analyzing the dielectric properties of polyurea with different chemical structures. Without the alkyl substituent chains in the polymer backbone, a high dielectric constant (*k*~5.8) was obtained by strengthening the hydrogen bonding interactions (Figure 3a). In addition, the leakage current density (*J*) was decreased because of the reduced free volume, resulting in low *J* even with 60 nm thickness (Figure 3b). Dinaphtho[2,3-*b*:2′,3′-*f*]thieno[3,2-*b*]thiophene (DNTT)-based organic FETs were fabricated using the polyurea dielectric layer, which showed low operating voltages (<3 V). The charge carrier *μ* was improved from 0.069 cm^2^·V^−1^·s^−1^ (untreated) to 1.390 cm^2^·V^−1^·s^−1^ (with the surface treatment layer) with the metal oxide-assisted surface treatment layer. Table 3 summarizes the key parameters of the FET devices based on polymer dielectric materials that are the focus of this paper.

Crosslinking is a powerful strategy to improve the dielectric strength of polymer dielectric materials by densifying the polymer matrices [2,5,80,114,115,116,117,118,119,120,121,122,123,124,125,126]. A previous study reported a reduced leakage current after a crosslinking structure was introduced in polymer dielectric layers [127,128]. Furthermore, Choi et al. investigated the effect of the chain length in the crosslinkers [5]. A monomer containing polar cyano group, CEA, was copolymerized with DEGDVE and 1,4-butanediol divinyl ether (BDDVE). These crosslinkers have similar chemical structures but different chain lengths. High-purity polymer films and homogenous mixing between CEA and crosslinker were achieved using the iCVD process to fabricate polymer dielectric layers. The decrease in dielectric constant according to the addition of a crosslinker was alleviated in poly(2-cyanoethyl acrylate-co-1,4-butanediol divinyl ether) [p(CEA-co-BDDVE)] compared with p(CEA-co-DEGDVE) (Figure 3c). Hence, with a similar crosslinker mole fraction, the copolymer dielectric with a short-chain crosslinker can exhibit a higher dielectric constant. 2,7-Dioctyl[1]benzothieno[3,2-*b*][1]benzothiophene (C8-BTBT)-based FETs were fabricated using p(CEA-co-BDDVE) with the optimal chemical composition, which showed a low operating voltage of less than 5 V and a high carrier level *μ* (>3.0 cm^2^·V^−1^·s^−1^). Moreover, the organic FETs were fabricated on a plastic substrate, maintaining their electrical characteristics even under the applied tensile strain up to ~2.4%.

Recently, intrinsically stretchable FET devices with highly deformable electronics, such as stretchable displays and electronic skins, have emerged [34,126,127,128,129,130]. For the reliable operation of stretchable FETs, intrinsically stretchable polymer dielectric materials have been widely researched, and crosslinking also plays a significant role in designing such materials [35,101,102,131,132,133,134,135,136,137,138,139]. Kang et al. developed a stretchable polymer dielectric composed of dicarboxy-terminated poly(acrylonitrile-co-butadiene) (CTBN) as a soft matrix and 1,6-bis(trichlorosilyl)hexane (C_6_) as a hard segment (Figure 3d) [101]. The leakage current was decreased as a portion of the C_6_ crosslinking agent increased. In particular, a metal–insulator–metal (MIM) device with CTBN:7wt.%-C_6_ maintained *J* of ~10^−8^ A·cm^−2^ even under an applied strain of 30%. Poly(3-hexylthiophene) nanowires (P3HT NWs)/polydimethylsiloxane (PDMS) blend organic semiconductor-based stretchable FETs were fabricated using a CTBN:7wt.%-C_6_ dielectric layer (Figure 3e). Although *μ* decreased with the applied strain, the stretchable FETs retained a gate leakage current of ~10^−9^ A, enabling FET devices with distinguishable on- and off-states.

In addition to the gate dielectric layer, polymer dielectric materials have been used as an additional layer on top of FETs to enhance the charge transport characteristics and the environmental stability of FET devices [104,140,141,142,143,144,145,146,147,148]. An electron-rich polyethylenimine (PEI) is a widely used n-doping polymer in metal oxide and 2-dimensional (2D) semiconductor FETs [149,150,151,152,153]. Hong et al. introduced truncated-branched PEI on top of 2D molybdenum disulfide (MoS_2_) semiconductor FET devices (Figure 3f) [150]. Compared to pristine 2D MoS_2_ FETs (~3.7 V), the PEI-doped device showed *V*_T_ = ~0.72 V, and this negative shift of *V*_T_ supported the strong n-doping effect of PEI (Figure 3g). Moreover, the on-current was increased from ~3.3 μA to ~4.8 μA, which could be explained by the reduced Schottky barrier and facilitated electron injection from the source to the channel layer with the PEI doping layer. In addition, 2D MoS_2_ FET with a PEI doping layer was used as a photodetector, which exhibited enhanced photo-responsivity compared to the pristine device. When light is irradiated, some of the holes in the photo-generated electron–hole pairs are trapped in a new energy level formed by PEI, leading to band-bending in the energy band and enhancing the photoresponsivity.

One of the noticeable issues in FETs with emerging semiconductor materials, such as organic and 2D semiconductors, is their susceptibility to ambient air, and hydrophobic polymers can be used as barrier layers [104,140,141,143,146,148]. Park et al. introduced a barrier polymer on top of nanostructured 2D MoS_2_ FETs [104]. The nanostructured 2D materials can exhibit unique electrical and optical properties by periodically removing the layer. On the other hand, they showed some disadvantages related to the lack of electrical stability resulting from the edge exposures. These issues were addressed by depositing poly(1H,1H,2H,2H-perfluorodecyl methacrylate) (pPFDMA) on nanoporous 2D MoS_2_ FETs by the iCVD process, which only exhibited a marginal change in the electrical characteristics of the FET devices owing to the solvent-free nature of the iCVD process. The hydrophobic pPFDMA effectively passivated nanoporous 2D MoS_2_ FETs and prevented moisture penetration. As a result, the ambient stability was improved remarkably in the pPFDMA-passivated nanoporous 2D MoS_2_ FETs compared to the pristine, non-passivated ones (Figure 3h). As extensively described above, the charge accumulation capability and dielectric reliability were significantly improved by enhancing the dielectric constant and/or introducing a crosslinking network. In addition to the gate dielectric layer, polymer materials can be used as functional adlayers to optimize the electrical characteristics and elevate the ambient stability of FET devices. Another effective strategy is utilizing polyelectrolytes and their composites to boost the charge transport performance and lower the power consumption of FETs, which also paves the way to develop FET-based sensors and neuromorphic devices [154,155,156,157,158,159]. Additionally, introducing ferroelectric polymers and incorporating inorganic components allow for further enhancing the performance of FETs or imparting unique behaviors, as described in the following sections.

**Table 3:** Summary of polymer gate dielectric-based FETs.

Dielectric Material	Semiconductor	Operating Voltage (V)	Current on/off Ratio	Mobility (cm^2^·V^−1^·s^−1^)	Subthreshold Swing (mV·dec^−1^.)	Applied Strain (%)	[Ref.]
Polyurea	DNTT	3	>10^5^	1.39	370	2	[97]
P(CEA-co-BDDVE)	C_8_-BTBT	5	>10^6^	3.39	87.4	2.5	[5]
CTBN-C6	P3HT NWs/PDMS	50	>10^4^	0.0031	N/A	34	[101]
	pentacene	50	>10^6^	0.12	N/A	N/A	
PVDF-TrFE (vertically poled)	TIPS-pentacene	4	>10^4^	0.08	200	N/A	[160]
PVDF-TrFE (textured poled)	TIPS-pentacene	4	>10^5^	1	350	N/A	
4FCOC ^1^	C_10_-DNTT	30	>10^7^	1.30	N/A	N/A	[161]
	PTCDI-C13 ^2^	30	>10^6^	0.13	N/A	N/A	
PVDF-TrFE	2D MoS_2_	40	>10^5^	N/A	24.2	N/A	[162]
HfO_2_/PVDF-TrFE	2D MoS_2_	2.5	>10^5^	N/A	42.5	N/A	[163]
PMMA/Al_2_O_3_	2D InSe	8	>10^7^	1055	300	N/A	[107]
P(CEA-co-DEGDVE)/Al_2_O_3_	C_8_-BTBT	3	>10^6^	2.48	68.4	1.2	[164]
CYTOP/HfO_2_-Al_2_O_3_	TIPS-pentacene/PTAA	10	>10^5^	0.8	700	N/A	[165]
	diF-TES-ADT ^3^/PTAA	10	>10^5^	1.4	320	N/A	
PSAND-4	pentacene	3	>10^3^	0.40	N/A	N/A	[106]
	F_16_CuPC ^4^	2	>10^1^	0.020	N/A	N/A	
	DPP-DTT	3	>10^3^	0.17	N/A	1.2	
	PDIF-CN_2_ ^5^	2	>10^2^	0.18	N/A	N/A	
BaTiO_3_/PDMS	Carbon nanotube	30	>10^2^	4	N/A	50	[166]

^1^ cyclo-olefin copolymer (COC) with fluorophenyl azide (FPA) crosslinker (4FCOC). ^2^ N,N’-Ditridecyl-3,4,9,10-perylenetetracarboxylic diimide (PTCDI-C13). ^3^ 2,8-difluoro-5,11-bis(triethylsilylethynyl) anthradithiophene (diF-TES-ADT). ^4^ 1,2,3,4,8,9,10,11,15,16,17,18,22,23,-24,25-hexadecafluoro-29H,31H-phthalocyanine (F_16_CuPc). ^5^ N,N′-1H,1Hperfluorobutyl dicyanoperylenecarboxydiimide (PDIF-CN_2_).

#### 3.2.3. Ferroelectric Polymers for FET Devices

**Figure 4:**
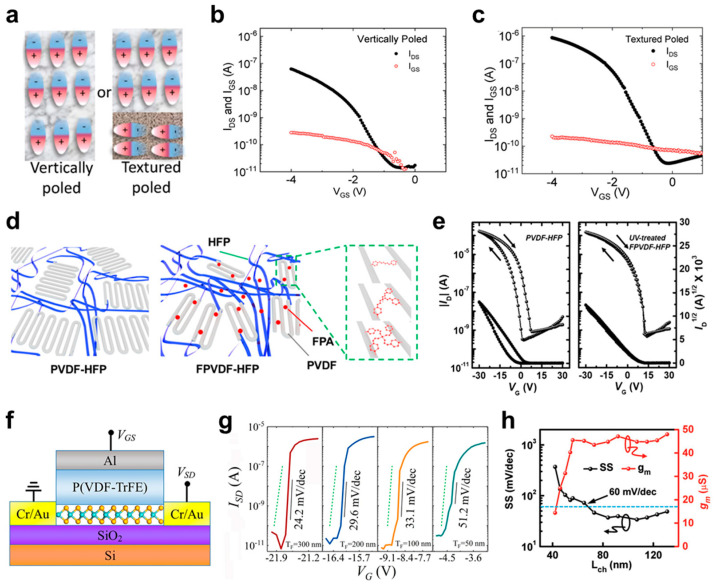
(**a**) Schematic diagram of vertically poled and textured poled P(VDF-TrFE). The transfer curves of TIPS-pentacene FETs with (**b**) vertically poled and (**c**) textured poled P(VDF-TrFE) as a gate dielectric layer [160]. Copyright © 2019, John Wiley and Sons. (**d**) The schematic diagram of PVDF-HFP and FDA-crosslinked PVDF-HFP (FPVDF-HFP). (**e**) The transfer curves of C_10_-DNTT organic FETs based on pristine PVDF-HFP and crosslinked FPVDF-HFP [161]. The arrows represent forward and backward sweep of *V*_G_. Copyright © 2020, American Chemical Society. (**f**) The schematic diagram of the device structure of the 2D MoS_2_ NC-FETs and (**g**) their transfer curves with different P(VDF-TrFE) thicknesses [162]. Copyright © 2017, Springer Nature. (**h**) The change in *SS* and *g*_m_ according to the channel length of 2D MoS_2_ NC-FETs based on HfO_2_/P(VDF-TrFE) gate dielectric layer [163]. Copyright © 2018, John Wiley and Sons.

PVDF-based ferroelectric polymers, such as poly(vinylidene fluoride trifluorethylene) (PVDF-TrFE) and poly(vinylidene fluoride-trifluoroethylenechlorofloroethylene) (PVDF-TrFE-CFE), have been investigated widely in FETs because of their high dielectric constant at room temperature [160,167,168,169,170,171,172,173,174,175,176,177,178,179]. Laudari et al. investigated the effect of poling in ferroelectric polymers using 6,13-bis(triisopropylsilylethynyl)pentacene (TIPS-pentacene) organic FETs to achieve reliable operation of FET devices based on ferroelectric polymer dielectrics [160]. The poling of the PVDF-TrFE was controlled by applying an external electric field during film crystallization, which enabled the fabrication of uniform, vertical poling, and textured poling (Figure 4a). Compared to the vertically poled PVDF-TrFE-based devices, TIPS-pentacene FETs with textured poled PVDF-TrFE exhibited an enhanced on-current and slightly decreased off-current (Figure 4b,c). Quantitatively, the textured poled PVDF-TrFE FET device showed the *μ* of ~1 cm^2^·V^−1^·s^−1^, current on–off ratio (*I*_on_/*I*_off_) of 10^5^, whereas vertically poled PVDF-TrFE FET showed *μ* of ~0.08 cm^2^·V^−1^·s^−1^ and 10^4^. The device performance was improved by an order of magnitude in textured poled PVDF-TrFE FETs while maintaining the low operating voltage of less than 4 V.

Blending and layer-by-layer structures have been proposed to improve the electrical and thermal properties of the ferroelectric polymer dielectrics [180,181,182,183,184,185,186,187]. Crosslinking is an efficient way to improve the electrical characteristics of the ferroelectric polymers and the resulting FET devices [161,188,189,190]. For example, Kwon et al. introduced fluorophenyl azide (FPA) to induce the crosslinking network in poly(vinylidene fluoride-co-hexafluoropropylene) (PVDF-HFP) (Figure 4d) [161]. A series of FPA was synthesized with a different number of fluorobenzene rings (2, 3, and 4), which can react with C−H bonds in conventional polymers under ultraviolet (UV) irradiation. Hysteresis in the transfer curves of 2,9-di-decyl-dinaphtho-[2,3-*b*:2′,3′-*f*]-thieno-[3,2-*b*]-thiophene (C_10_-DNTT) organic FETs disappeared through FPA crosslinking (UV-treated FPVDF-HFP) (Figure 4e). In the PVDF-based ferroelectric polymer dielectric, carbon (C)−fluorine (F) dipoles were rearranged under a high electric field, enabling ferroelectric behavior. This rearrangement of C−F dipoles remained even after the applied electric field was removed, causing the clockwise hysteresis in the current−voltage (*I*−*V*) characteristics of FET devices. On the other hand, with the addition of the FPA crosslinker, the grain size of the VDF crystals and the number of ferroelectric domains were decreased, reducing the hysteresis behavior of the FET devices.

NC behavior, which achieves a lower subthreshold swing (*SS*) than the theoretical limit at room temperature (~60 mV·dec^−1^.), is another attractive property of ferroelectric polymer dielectric materials [162,163,191,192,193,194]. Wang et al. developed NC-FETs based on 2D MoS_2_ and ferroelectric P(VDF-TrFE) (Figure 4f) [162]. The thickness of P(VDF-TrFE) in 2D MoS_2_ NC-FETs was varied from 50 to 300 nm and all the devices exhibited *SS* values lower than 60 mV·dec^−1^. (Figure 4g). In particular, the *SS* values decreased gradually as the thickness of P(VDF-TrFE) increased because there was a certain layer at the interface between P(VDF-TrFE) and the top Al electrode that barely contributed to the NC behavior. This was confirmed by linear fitting, where a fixed capacitance of 5.5 nF was observed even when the thickness of P(VDF-TrFE) was projected to be zero. Nevertheless, the ultralow *SS* value of ~24.2 mV·dec^−1^. was obtained with a thick P(VDF-TrFE) dielectric layer (~300 nm). Liu et al. also developed NC-FETs using 2D MoS_2_ and P(VDF-TrFE), and 4 nm-thick HfO_2_ was inserted between 2D MoS_2_ and P(VDF-TrFE) to achieve the reliable NC behavior and robust operation of FETs [163]. Furthermore, silver nanowires (AgNWs) were used as gate electrodes to reduce the channel length and maximize the output current of the NC-FETs. An *SS* value lower than 60 mV·dec^−1^. and high transconductance (*g*_m_) were maintained with channel lengths greater than 60 nm (Figure 4h). With further decreases in channel length, *SS* increased, and *g*_m_ decreased because of the short channel effects. Nevertheless, this study showed the great potential of 2D channel material and ferroelectric polymers in high-performance nano-scale electronics.

#### 3.2.4. Polymer–Inorganic Hybrid Dielectric Materials for FET Devices

**Figure 5:**
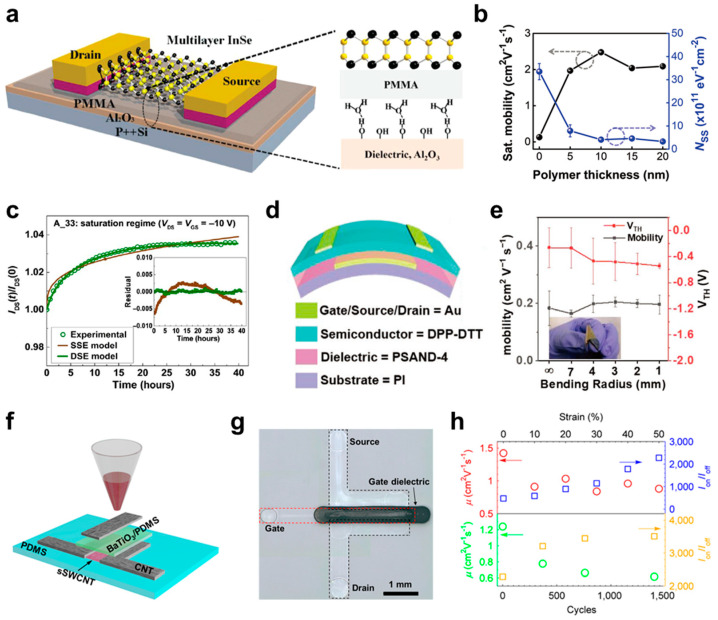
(**a**) Schematic diagram of the 2D InSe FET with a PMMA/Al_2_O_3_ gate dielectric layer [107]. Copyright © 2014, John Wiley and Sons. (**b**) Change in the *μ* and *N*_SS_ of C8-BTBT FETs according to the thickness of the polymeric layer in p(CEA-co-DEGDVE)/Al_2_O_3_ hybrid dielectric [164]. Black and blue lines represent mobility and trap density, respectively. Copyright © 2022, John Wiley and Sons. (**c**) *I*_D_ change with respect to the bias stress time of TIPS-pentacene/PTAA FETs with the CYTOP/HfO_2_-Al_2_O_3_ dielectric layer [165]. Copyright © 2018, American Association for the Advancement of Science. (**d**) The schematic diagram of flexible DPP-DTT FETs based on PSAND-4 dielectrics and (**e**) their mobility and *V*_T_ change according to the bending radius [106]. Copyright © 2020, John Wiley and Sons. (**f**) The schematic diagram of the fabrication process of SWCNT FETs with the BaTiO_3_/PDMS composite dielectric layer and (**g**) the optical microscopy image of the fabricated device. (**h**) The change in *μ* and *I*_on_/*I*_off_ with respect to the applied strain and strain cycles of SWCNT FETs [166]. Red and blue dots represent mobility and *I*_on_/*I*_off_ in the upper graph, respectively, and green and yellow dots represent mobility and *I*_on_/*I*_off_ in the lower graph, respectively. Copyright © 2016, American Chemical Society.

Organic–inorganic hybrid dielectric materials have been developed to accommodate the advantages of organic and inorganic materials, and polymers have been incorporated as organic components in hybrid dielectrics [100,195,196]. For example, polymer dielectric layers have been inserted between inorganic dielectric layers and semiconductors to passivate interface traps of the inorganic layers [107,164,197,198,199,200,201,202]. Feng et al. used poly(methyl methacrylate) (PMMA)/Al_2_O_3_ bilayer dielectrics for 2D indium selenide (InSe) FET devices (Figure 5a) [107]. The PMMA layer passivated the hydroxyl (−OH) groups of Al_2_O_3_ and suppressed charge carrier scattering, which was supported by analyzing the performance of FET. The *μ* value of the 2D InSe FET with the Al_2_O_3_ dielectric layer was ~64 cm^2^·V^−1^·s^−1^, which was enhanced drastically to ~1055 cm^2^·V^−1^·s^−1^ with the PMMA layer. In addition, the *SS* value was improved from 600 to 300 mV·dec^−1^. by inserting PMMA. This enhancement in the FET performance was consistent when SiO_2_ replaced Al_2_O_3_ as the inorganic dielectric layer, and the increase in the *μ* value was commonly obtained with different thicknesses of 2D InSe (10–50 nm). These results suggested that the Coulomb impurities or surface polar phonon scattering can be suppressed by the polymer dielectric layer. Similarly, Choi et al. developed polymer–inorganic hybrid dielectrics consisting of plasma-grown Al_2_O_3_ and high-*k* p(CEA-co-DEGDVE) [164]. The polymeric layer thickness was adjusted precisely on the nanometer scale by tuning the deposition time in the iCVD process. *μ* was increased, and the interface trap density (*N*_SS_) was decreased in C8-BTBT organic FETs by introducing the polymer dielectric layer (Figure 5b). The thickness of the polymer dielectric was optimized to 10 nm to achieve high-performance FET devices and reliable dielectric properties, and the total thickness of the layered hybrid dielectric was 15 nm (5 nm-thick Al_2_O_3_ and 10 nm-thick high-*k* polymer). Based on this high dielectric constant and ultralow thickness, low-voltage (<3 V), flexible C8-BTBT organic FETs were demonstrated, which exhibited 100% yield and a high areal uniformity in the FET characteristics.

FET devices with high operational stability were achieved by exploiting the reduced trap densities of layered hybrid dielectrics [165,200,203,204,205,206,207]. Jia et al. fabricated TIPS-pentacene/poly[bis(4-phenyl) (2,4,6-trimethylphenyl) amine] (PTAA) organic FETs and 35 nm-thick CYTOP and 33 nm-thick HfO_2_-Al_2_O_3_ nanolaminate were used as a gate dielectric layer [165]. Extremely high operational stability was achieved in the organic FETs where the change in *I*_D_ was less than 4% of the initial *I*_D_ even after 40 h of the continuous bias stress, which was well-matched with modeling results using double-stretched exponential (DSE) function (Figure 5c). This long-term bias stress stability could be explained by two competing processes, where *I*_D_ decreased by charge trapping at the interface between the semiconductor and polymer dielectric layer, being compensated by remnant dipole polarization in the inorganic layer [164,208]. In addition, the FETs were fabricated in top-gate geometry, allowing for an improved environmental stability.

Incorporating polymer dielectric materials into hybrid dielectrics can improve mechanical flexibility by mitigating the brittleness of inorganic dielectric materials [4,106,209,210,211]. For example, Chen et al. developed polymeric self-assembled nanodielectric (PSAND) by the solution-processed layer-by-layer assembly of a P-PAE polymer chromophore and zirconia (ZrO_x_) [106]. The thickness of the hybrid insulating layer was adjusted precisely according to the assembly frequency (PSAND-x), and the fabricated hybrid insulating layer exhibited high capacitance (558, 472, 406, and 339 nF·cm^−2^ for PSAND-1, 2, 3, and 4, respectively). In particular, the *C*_i_ value varied linearly with the PSAND thickness, indicating a well-defined multilayer structure. Diketopyrrolopyrrole-dithienyl-thieno[3,2-*b*] thiophene (DPP-DTT) organic FETs were fabricated on polyimide (PI) substrates using the PSAND-4 as a dielectric layer (Figure 5d), with an operating voltage less than 3 V enabled by high *C*_i_ of PSAND-4. The fabricated flexible organic FETs only exhibited a marginal change in their *μ* and *V*_T_ with the bending radius from ∞ to 1 mm, showing good mechanical flexibility of the self-assembled hybrid dielectrics.

A nanocomposite is another type of polymer–inorganic hybrid dielectric material, where inorganic nanoparticles are dispersed within a polymer matrix [166,212,213,214,215,216]. Cai et al. developed fully printable, semiconducting single-walled carbon nanotube (SWCNT) FETs using the nanocomposite hybrid dielectrics consisting of barium titanate (BaTiO_3_) nanoparticles and PDMS (Figure 5f,g) [166]. Unsorted carbon nanotubes (CNTs) were used as gate and source/drain electrodes, of which the resistance decreased significantly as the number of printing cycles increased. The dielectric constant of the BaTiO_3_/PDMS composite increased with the BaTiO_3_ volume content increased, and it reached ~9 at a BaTiO_3_ volume content of ~26%. A *C*_i_ value of ~2.8 nF·cm^−2^ was obtained, even with a thickness of ~2 μm owing to the high dielectric constant. The composite dielectric layer exhibited robust insulating properties with *J* < 10^−8^ A·cm^−2^ under a voltage as high as ±200 V. Moreover, the *C*_i_ values of the BaTiO_3_/PDMS composite showed only a marginal change under the repeated stretching with the applied strain of 50% for 2000 cycles. This excellent mechanical property made it possible to fabricate stretchable SWCNT FETs on a PDMS substrate, which retained its FET operation under an applied strain of 50% and under the 1400 cycles of repeated stretching with 50% strain. The *μ* value tended to decrease, and *I*_on_/*I*_off_ increased according to the applied strain and strain cycle, possibly due to the structural changes in the SWCNT network and the stronger gating effect, respectively (Figure 5h). Furthermore, stretchable logic circuits, including NOR and NAND gates, were demonstrated by integrating FET devices.

In addition to the hybrid dielectrics described above, homogeneous polymer–inorganic hybrid dielectric materials were developed using solution-phase fabrication [108,217,218,219] and vacuum deposition [109,220,221] to improve the electrical and mechanical properties of the dielectric layers.

### 3.3. Triboelectric Nanogenerators (TENGs)

#### 3.3.1. Overview of Polymer Dielectric Materials for TENGs

Various types of dielectric materials have been introduced to accommodate the important requirements of TENGs, including high output performance, low operating voltage, and device stability. Directly injecting electric charge into the surface of a dielectric layer significantly improves the surface charge density and enhances the output performance of TENGs, adding a gradient charge-confinement layer containing mesoporous carbon spheres, further boosting these parameters [222,223]. Similarly, using the self-assembled monolayer (SAM) method for the atomic-level chemical functionalization of polymer surfaces significantly alters the dielectric properties and enhances the triboelectric performance of TENGs [224,225]. In addition, incorporating metal oxide nanoparticles into the triboelectric layer increases the dielectric constant and surface charge density, further improving the output performance of TENGs [226,227]. Dissolving P(VDF-TrFE) in high dipole moment solvents enhances the polymer chain length, crystallinity, and dipole alignment, leading to superior triboelectric properties and improved TENG output performance [228,229].

Moreover, the application of electrical polarization to ferroelectric composite layers by controlling the electric field, temperature, and process time improves the ferroelectric properties and allows the precise modulation of charge transfer and output voltage in TENGs [230,231]. Furthermore, multilayered PVDF-TrFE/BaTiO_3_ films with aligned BaTiO_3_ nanoparticles significantly enhance the dielectric constant, local field, and polarization, resulting in a higher output current density and improved TENG performance [27]. The subsequent section will review the latest research breakthroughs in enhancing polymer-based dielectric materials specifically for TENGs. Table 4 provides a summary of the parameters for the TENG devices that utilize polymer dielectric materials, which are the focus of this paper.

#### 3.3.2. Polymer-Based Dielectric Materials and Strategies for Enhancing TENG Devices

**Figure 6:**
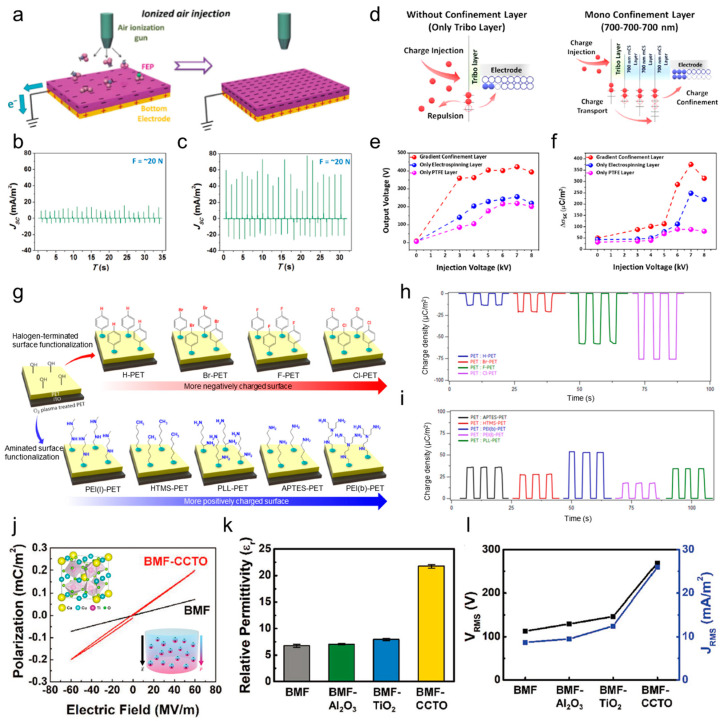
(**a**) Schematic diagram of the basic ion injection process onto the FEP film. The short-circuit current density of the TENG (**b**) before and (**c**) after the injection process. Reprinted with permission from [243], Copyright 2014, Wiley-VCH. (**d**) The schematic diagram of charge transport and confinement mechanisms in different charge confinement layers. (**e**) The comparison of the output voltages based on the gradient arrangement of mCSs. (**f**) The comparison of the surface charge densities based on the gradient arrangement of mCSs. Reprinted with permission from [223], Copyright 2022, Wiley-VCH. (**g**) The schematic diagram of surface-functionalized negative and positive PETs with adopted molecules. (**h**,**i**) The surface charge density for various functional groups. Reprinted with permission from [244], Copyright 2017, American Chemical Society. (**j**) The *P*–*E* curves of pure BMF and BMF–CCTO 1 wt.% composite material (inset: chemical structure of CCTO and the relationship between relative permittivity and polarization under an electric field). (**k**) The relative permittivity of pure BMF, BMF–Al_2_O_3_, BMF–TiO_2_, and BMF–CCTO composite materials (1 wt.%). (**l**) The *V*_RMS_ and *J*_RMS_ of pure BMF, BMF–Al_2_O_3_, BMF–TiO_2_, and BMF–CCTO (1 wt.%) composite materials in rotation-type freestanding mode TENGs. Reprinted with permission from [60], Copyright 2020, Wiley-VCH.

Directly injecting electric charge into the surface of a dielectric layer is a straightforward yet highly effective method for greatly increasing surface charge density. Wang et al. demonstrated that the output performance of TENGs could be enhanced by using ionized air to inject charges into a fluorinated ethylene propylene (FEP) film with an air ionization gun (Figure 6a) [232]. The electret film created through ion implantation maintained a surface charge density (Δ*σ*) of approximately 200 μC·m^−2^ for 160 days, with a minimal loss rate of 16.6%. The TENG with an ionized FEP film exhibited a five-fold increase in the short-circuit current density compared to the untreated FEP film (Figure 6b,c).

In another study, Cha et al. introduced a charge injection process to a gradient charge-confinement layer composed of electrospun nanofibers to enhance the surface charge density of the dielectric layer [233,234,235]. Each successive nanofibrous layer, from the innermost to the outermost, contained increasingly larger amounts of mesoporous carbon spheres (mCS), as illustrated in Figure 6d. These mCSs facilitated the transport of charges from the outer surface, influenced by the charge injection, to the deeper regions of the nanofibrous layer while also minimizing charge loss through confinement. This gradient arrangement of mCSs resulted in a high space charge density within the charge-injected composite layer (Figure 6e). When the charge injection was carried out under a strong external field of 7 kV, the surface charge density in the gradient charge-confinement layer was approximately 7.5 times greater than in the absence of mCSs. As a result, the output voltage surged dramatically to 600 V after the high-voltage charge injection compared to just 15.2 V before the injection (Figure 6f). In summary, the combination of the charge injection with the strategic structural design of the dielectric layer presents an effective approach for creating high-performance TENGs.

The SAM method [225,236,237], which relies on the spontaneous organization of chemisorbed surfactant molecules on solid surfaces, serves as another effective method for chemical surface modification. Shin et al. introduced a straightforward SAM approach to achieve atomic-scale chemical functionalization aimed at modifying the dielectric properties of polymer surfaces (Figure 6g). In their study, SAMs were methodically formed on a polyethylene terephthalate (PET) substrate through the sequential application of various halogens and amines. The process began with the functionalization of the PET surface using hydroxyl groups (−OH) via oxygen plasma treatment, creating an intermediate layer that promotes chemical bonding with desired functional molecules. Subsequently, this hydroxyl-coated layer was treated with halogen-terminated (Br, F, and Cl) phenyl derivatives, acting as tribo-negative materials, and different amine compounds, serving as tribo-positive materials, to impart negative or positive triboelectric characteristics to the PET substrate accordingly. The resulting aminated PET substrates demonstrated strong tribo-positive behavior, while the halogenated PET substrates exhibited pronounced tribo-negative properties. Constructing TENGs by pairing samples with opposing triboelectric polarities, such as PEI(b)-PET and Cl-PET, yielded high voltage outputs of 300 V and 200 V for each layer, respectively. Overall, this atomic-level chemical functionalization technique provides a simple and efficient strategy to enhance the design versatility of polymer substrates in the development of high-performance TENGs.

Another effective strategy for achieving high-performance TENGs is to directly adjust the intrinsic dielectric constant of the active triboelectric layer, which maximizes dielectric polarization across the triboelectric layers. This can be accomplished by creating a triboelectric layer in the form of a nanocomposite that incorporates high-k metal oxide nanoparticles (such as BaTiO_3_, BiFeO_3_, and CCTO) [238,239,240,241] dispersed within a ferroelectric polymer matrix, thereby effectively increasing the dielectric constant. These high-k nanoparticles boost the surface charge density of the triboelectric layer, which in turn enhances the output performance of TENGs.

Kim et al. developed a composite triboelectric layer composed of butylated melamine formaldehyde (BMF) and high-dielectric CaCuTi_4_O_12_ (CCTO) nanoparticles designed to produce a high-output TENG with consistent performance (Figure 6j–l). The CCTO nanoparticles, with a high dielectric constant of 7500, induced substantial dielectric polarization in the composite triboelectric layer, leading to more efficient charge induction at the counter electrode and boosting the TENG’s output performance. The dielectric constant of the BMF-CCTO composite layer (at 1 wt.%) was measured to be 21.74, which was approximately three times higher than that of pristine BMF, BMF-Al_2_O_3_, and BMF-TiO_2_. The relationship between permittivity and electrical polarization can be expressed as

(6) $$\vec{P}=\varepsilon_{0}\left(\varepsilon_{r}-1\right)\vec{E}$$

where $\vec{P}$, $\varepsilon_{r}$, and $\vec{E}$ are the electrical polarization within a material, relative permittivity, and applied electric field, respectively [242]. According to Equation (6), a higher dielectric constant leads to stronger internal polarization when subjected to an electric field. Incorporating 1 wt.% CCTO into BMF generated a voltage of 268 V and a current density of 25.8 mA/m^2^ [60]. In addition, ferroelectric materials were used to alter dipole alignment, which further boosted the performance of TENGs. The described advancements in enhancing the performance of TENGs highlight the significance of combining innovative charge injection techniques with material engineering. The use of ionized air, gradient charge-confinement layers, and high-dielectric materials demonstrates the potential for significantly increasing surface charge density and output efficiency. These approaches not only push the boundaries of TENG technology but also open up new possibilities for designing high-performance energy-harvesting devices. It could also be broadly applicable to other dielectric-based devices, such as capacitors or sensors, where maximizing charge storage and dielectric polarization is essential.

#### 3.3.3. Ferroelectric Polymers for TENG Devices

**Figure 7:**
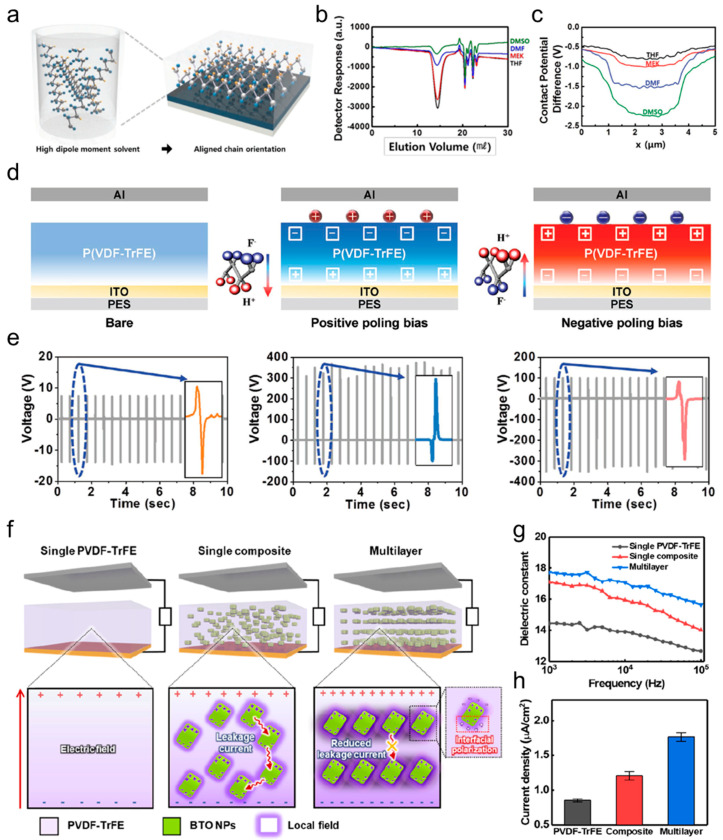
Schematic diagram of the ferroelectric P(VDF-TrFE) to modulate output performance and related methods. (**a**) The schematic diagram of P(VDF-TrFE) dissolved in a high dipole moment solvent and the resulting P(VDF-TrFE) film. (**b**) The GPC measurement of the molecular weight (*M*_w_) of P(VDF-TrFE) dissolved in four solvents (THF, MEK, DMF, and DMSO); a higher *M*_w_ indicates a longer chain length of P(VDF-TrFE). (**c**) The CPDs of P(VDF-TrFE) dissolved in the four solvents; a bias was applied to the central area of P(VDF-TrFE) for poling. Reproduced from [247], Copyright 2017, WILEY-VCH Verlag GmbH & Co. KGaA, Weinheim. (**d**,**e**) Controllable charge transfer through ferroelectric polarization. Reproduced from [252], Copyright 2016, WILEY-VCH Verlag GmbH & Co. KGaA, Weinheim. (**f**) The schematic diagram of three different sample types with the same thickness: a PVDF-TrFE film, a single PVDF-TrFE/BTO composite, and a multi-layered PVDF-TrFE/BTO film. (**g**) The comparison of the dielectric constants of the three different types of films. (**h**) The comparison of the output current density under a vertical pressure of 98 kPa at 2 Hz. Reproduced from [250], Copyright 2020, American Chemical Society.

Figure 7a shows P(VDF-TrFE) dissolved in a solvent with a high dipole moment, leading to longer polymer chain lengths, improved chain orientation, and increased crystallinity. The enhanced crystallinity of P(VDF-TrFE) in these solvents is mainly attributed to the extended end-to-end chain length of the polymer [245,246]. Kim et al. determined the *M*_w_ values using gel permeation chromatography (GPC) because the relative chain length of P(VDF-TrFE) can be expressed in terms of the weight average molecular weight (*M*_w_). The GPC of P(VDF-TrFE) dissolved in tetrahydrofuran (THF), methyl ethyl ketone (MEK), dimethylformamide (DMF), and dimethyl sulfoxide (DMSO) revealed 2.37 × 10⁵, 2.44 × 10⁵, 2.75 × 10⁵, and 2.76 × 10⁵ g·mol^−1^, respectively (Figure 7b). These results confirm that P(VDF-TrFE) dissolved in higher dipole moment solvents (e.g., DMSO) has a higher *M*_w_ and a longer chain length. The alignment of dipoles plays a crucial role in the performance of ferroelectric polymer-based TENGs, as greater dipole alignment in the polymer can lead to increased output. To evaluate improvements in energy harvesting, the triboelectric properties of P(VDF-TrFE) dissolved in four different solvents were analyzed. Figure 7c illustrates the contact potential differences (CPDs) of P(VDF-TrFE) in these solvents, measured using Kelvin probe force microscopy (KPFM). The CPDs between the P(VDF-TrFE) and the Pt tip were −0.87, −1.10, −1.63, and −2.37 V for THF, MEK, DMF, and DMSO, respectively. Based on these CPD measurements, the corresponding work functions of P(VDF-TrFE) dissolved in THF, MEK, DMF, and DMSO were calculated to be 5.91, 6.14, 6.67, and 7.41 eV, respectively. Consequently, P(VDF-TrFE) dissolved in a solvent with a higher dipole moment exhibits a more negative CPD and a larger work function, suggesting a greater capacity to attract electrons during triboelectrification and enhanced triboelectric properties [247].

Beyond controlling chain length, ferroelectric composite layers can enhance the ferroelectric properties of friction layers by triggering dipole rearrangement through the application of external electric fields [230,248]. Achieving the effective polarization of each composite requires optimizing factors such as the electric field, temperature, and processing time, which are influenced by the material’s coercive field, insulation resistance, and Curie temperature [249]. Additionally, the polarization direction must be carefully selected to strengthen the polarity of the friction surface (Figure 7d). The output voltages of positively poled P(VDF-TrFE)-based TENGs were opposite in sign to those of negatively poled P(VDF-TrFE)-based TENGs, suggesting that the polarization direction influences the sign of charge transfer during the triboelectric phenomenon.

Moreover, although the bare P(VDF-TrFE) device showed an output voltage of approximately 15 V, positively and negatively poled P(VDF-TrFE)-based TENGs under a vertical compressive force of 2 kg exhibited output voltages of up to 400 V and 100 V, respectively. These values are significantly higher than those of the bare P(VDF-TrFE)-based TENGs. This can be explained by the incomplete switching of the bare P(VDF-TrFE) in one direction. The varying output voltages also confirmed that the amount of charge transfer can be controlled by adjusting the polarization states. Figure 7d,e show that the sign and amount of charge transfer can be controlled by the polarization states.

Park et al. evaluated the triboelectric performance of three different film types to assess the impact of BaTiO_3_ (BTO) nanoparticle (NP) interlayers on triboelectric properties: a single PVDF-TrFE film, a single PVDF-TrFE/BTO nanocomposite, and multilayered PVDF-TrFE/BTO films (Figure 7f). The distribution of the electric field indicated that the multilayer structure, with BTO NPs arranged on the coplanar layer, offers more efficiently connected interfacial charges at closer distances than composites with randomly dispersed BTO NPs. This arrangement leads to a significantly enhanced local electric field and a greater ferroelectric polarization of the polymer [250].

Simulations using COMSOL Multiphysics revealed that the interfacial polarization in the multilayer structure (8.4 mC·m^−2^) was greater than in the single composite (8.06 mC·m^−2^). This increased polarization contributes to a higher dielectric constant in the multilayered film. Figure 7g illustrates how the dielectric constant varies with frequency across different films. While the PVDF-TrFE/BTO films exhibited a higher dielectric constant (15.9 at 10 kHz) compared to the PVDF-TrFE films (13.9 at 10 kHz) due to the presence of high-k BTO nanoparticles, the multilayered PVDF-TrFE/BTO film achieved the highest dielectric constant (17.06 at 10 kHz), attributed to the alignment and closer spacing of BTO nanoparticles in the coplanar layer. The polymer interlayers between the inorganic nanoparticle layers in these multilayered films also enhanced breakdown strength by reducing leakage current in the insulating polymer layers [251], resulting in superior dielectric properties compared to single composites. Figure 7h displays the average output current density for PVDF-TrFE and PVDF-TrFE/BTO films in both the single composite and multilayer configurations. The multilayered PVDF-TrFE/BTO films demonstrated an output current density of 1.77 μA·cm^−2^, which is 1.5 times higher than that of the composite film without a multilayer structure (1.2 μA·cm^−2^). The enhanced output performance of the multilayered PVDF-TrFE/BTO films, in comparison to single composite films, is due to the alternating PVDF-TrFE/BTO layer structure, which increases stress-induced polarization. Furthermore, the stronger local field generated by effective interfacial polarization within the multilayered PVDF-TrFE/BTO film also played a significant role in boosting the output performance.

Overall, electrically polarizing ferroelectric composite materials allows for the adjustment of the dielectric properties and the triboelectric friction surface, thereby enhancing TENG performance. Moreover, the polarized dipoles within the composite layer influence charge carrier movement, leading to an additional increase in the triboelectric output [252,253,254]. The findings highlight the importance of solvent dipole moments and controlled polarization in optimizing the dielectric properties and output performance of ferroelectric polymers in TENGs. By carefully selecting solvents and employing strategic polarization techniques, the molecular structure and dipole alignment of materials like P(VDF-TrFE) can be precisely controlled, leading to substantial enhancements in triboelectric performance. These insights extend beyond TENGs, offering a pathway for improving other dielectric-based devices where charge carrier movement and dielectric constant optimization are important, such as in capacitors and memory devices. The demonstrated benefits of multilayered structures and aligned nanoparticles suggest that similar strategies could be employed to enhance the efficiency and performance of a wide range of dielectric materials and devices.

#### 3.3.4. Polymer–Inorganic Hybrid Dielectric Materials for TENG Devices

Beyond surface and bulk modifications, engineering intermediate layers presents a promising strategy for enhancing triboelectric performance. These interlayer modifications are introduced between the triboelectric materials and electrodes, allowing for control over the interfacial properties based on the physical attributes of the interlayer materials. Two primary challenges include surface charge decay and the hindrance of electrostatic induction due to charge recombination at the electrodes [255]. Surface charges in contact with air can gradually dissipate because their electric field attracts ions that are constantly present in the atmosphere [256]. Furthermore, surface charges can decrease due to a drift process caused by the electric field and a diffusion process driven by the electron concentration gradient. This reduction in surface charges leads to a decline in the triboelectric performance. Electrostatic induction can also be hindered at the interface between the triboelectric materials and electrodes, where the presence of numerous free electrons in the electrodes may neutralize electrostatic induction through recombination [257]. The suppression of electrostatic induction has a detrimental effect on triboelectric behavior, resulting in lower performance. To address these challenges and enhance triboelectric efficiency, various interfacial engineering techniques have been thoroughly researched. This section examines interfacial engineering strategies, highlighting two primary approaches: electron-trapping layers (ETLs) and electron-blocking layers (EBLs).

**Figure 8:**
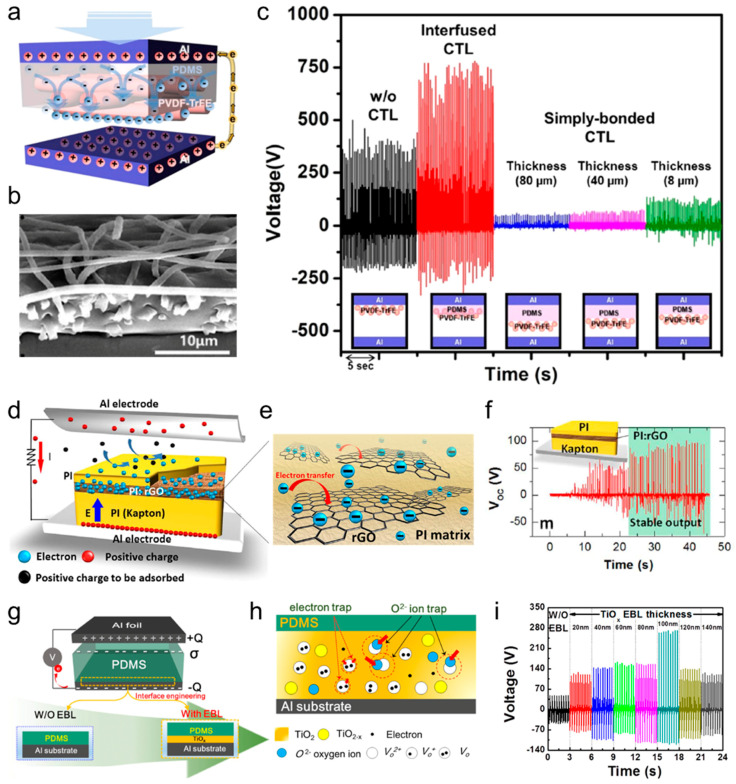
The designs of charge-trapping layers and their impact on triboelectric performance. (**a**) The illustration of the TENG device featuring an interfused charge-trapping layer. (**b**) The FE-SEM results of an electrospun PVDF layer and a PVDF/PDMS interfused friction layer. (**c**) The dependency of the thickness of the interlayer and configuration on the output voltage. Reprinted with permission from [260], Copyright 2022, Elsevier. (**d**,**e**) The schematic diagram of the PI/rGO multilayer TENG. (**f**) The measured open-circuit voltages (*V*_OC_) of the TENG based on the PI/rGO multilayer. Reproduced from [261], Copyright 2017, Elsevier. (**g**) The device design of an interface-engineered TENG based on the intermediate layer (TiO_x_) and (**h**) the description regarding the electron block event by the TiO_x_ interlayer. (**i**) Measured voltages as a function of the thickness of the intermediate layer. Reprinted with permission from [257], Copyright 2018, Elsevier.

Kim et al. introduced an innovative design for a TENG incorporating a polymeric interlayer. In this study, electrospun PVDF-TrFE served as the negative tribo-material, while PDMS was used as the charge-trapping layer [258,259] (Figure 8a). The design featured an interfused charge-trapping layer intended to capture triboelectric charges within the PDMS interlayer (Figure 8b). This interfused structure improved the contact between PVDF-TrFE and PDMS, thereby enhancing charge transfer from PVDF-TrFE to PDMS. As a result, the charge-trapping efficiency of the PDMS layer was significantly increased, leading to a triboelectric performance boost of approximately two times compared to a PVDF-TrFE-based TENG and six times higher than a TENG with a simply bonded PVDF-TrFE/PDMS structure (Figure 8c).

**Table 4:** Summary of polymer dielectric-based TENGs.

Dielectric Material	Working Mode	Surface Charge Density	Output Voltage	Output Current	Output Power	Applied Force, Pressure	[Ref.]
FEP	Single electrode	~200 μC·m^−2^	~1000 V_PP_	~78 mA/m^2^ (I_PP_)	315 W/m^2^	20 N	[243]
mCSs ^1^	Single electrode	~75 μC·m^−2^	~600 V_PP_	12.8 μA (I_PP_)	5.83 mW	80 N	[223]
PEI(b)-PET ^2^	Double electrode	52 μC m^−2^	~520 V_PP_	110 mA/m^2^ (I_PP_)	55 W/m^2^	0.15 MPa	[244]
BMF-CCTO ^3^	Free-standing	N/A	268 V_RMS_	25.8 mA/m^2^ (I_RMS_)	25.8 W/m^2^	N/A	[60]
PVDF-TrFE (DMSO)	Double electrode	N/A	~340 V_PP_	~220 μA (I_PP_)	N/A	1 kgf	[247]
PVDF-TrFE (poled)	Double electrode	20.86 nC·m^−2^	~400 V_amp_	N/A	N/A	1 kgf	[252]
PVDF-TrFE/BTO	Double electrode	N/A	~44 V_amp_	1.77 μA/cm^2^ (I_amp_)	29.4 μW/cm^2^	98 kPa	[250]
PVDF-TrFE/PDMS	Double electrode	N/A	~40 V_PP_	~350 μA (I_PP_)	125.5 mW/cm^2^	N/A	[260]
PI/rGO ^4^	Double electrode	N/A	190 V_amp_	~70 μA (I_amp_)	6.3 W/m^2^	N/A	[261]
PDMS/TiO_2_	Double electrode	30 μC·m^−2^	272 V_amp_	~9.1 μA (I_amp_)	N/A	5 N	[257]

^1^ mesoporous carbon spheres (mCSs). ^2^ branched polyethylenimine (PEI(b)-PET) functionalized on the O_2_ plasma-treated PET. ^3^ butylated melamine formaldehyde (BMF)–CaCu_3_Ti_4_O_12_ (CCTO). ^4^ reduced graphene oxide (rGO) on PI.

Researchers have investigated advanced charge-trapping layers made from conductive materials and polymer composites. Wu et al. demonstrated improved triboelectric performance using a reduced graphene oxide (rGO)-based electron-trapping layer (ETL) (Figure 8d). Polyimide (PI) was employed as the negative tribo-material, with a PI/rGO composite layer integrated within the PI layer to function as an electron storage layer. Additionally, rGO adjusted the interfacial energy band alignment (Figure 8e), enhancing its ability to trap electrons. Electron trapping resulted in more than three times improvement in the triboelectric performance compared to a pristine PI-based TENG device (Figure 8f).

Electron-blocking layers (EBLs) have been researched as a means to reduce electrostatic induction hindrance at the interface between triboelectric materials and electrodes. Park et al. proposed an EBL by incorporating a sputtered titanium oxide (TiO_x_) layer (Figure 8g). PDMS served as the friction layer and an Al film as the electrode in this setup. The electron-blocking mechanism relies on the capacity of oxygen vacancies in the TiO_x_ layer to capture free electrons (Figure 8h). These oxygen vacancies, which act as electrically positive defects, can trap free electrons from the Al electrode when an external electric field is applied. The efficiency of the TiO_x_ layer in blocking electrons is closely tied to the quantity of oxygen vacancies, highlighting the importance of increasing the ratio of these vacancies in the interlayer. Moreover, the interlayer affects both the electron-blocking capabilities and the device’s capacitance. As the material thickness increases, both the capacitance and electrostatic induction may decrease, making it crucial to achieve an optimal thickness for the interlayer. A 25-fold enhancement in the triboelectric performance was achieved using the electron-blocking effect (Figure 8i).

## 4. Challenges

### 4.1. Challenges in the Area of Dielectric Polymers for Future Memory Technologies

Polymer dielectric materials have been used extensively in memory devices, specifically in developing conductive filament memristors and charge-trap memory transistors. These applications leverage the unique electrical properties, processability, and flexibility of polymers to enhance device performance. On the other hand, several challenges and opportunities exist for polymer dielectric materials in memory applications:

(1) Electrical Property Improvement: Polymers have shown high dielectric constants and reliable insulating properties, but their electrical characteristics are often not as advanced as those of inorganic materials. Enhancing the dielectric constant and minimizing the leakage current paths, even at extremely thin thicknesses, requires molecular-level material and structural design. For example, incorporating polar functional groups that respond effectively to electric fields can improve the dielectric constant [262,263]. In addition, forming a denser polymer matrix can help reduce the leakage currents [97].
(2) Process Optimization: Polymer dielectric films are primarily fabricated through solution-based processing methods, which offer simplicity and potential for large-scale production [96,264]. On the other hand, these methods can introduce residual solvents or additives that adversely affect electrical properties [80]. Exploring alternative processing techniques, such as chemical vapor deposition (CVD), can help achieve high-purity polymer films and homogeneous mixing, improving the overall performance of memory devices [51,55,85,86,95].

Despite these challenges, the flexibility, tunability, and ease of the processing of polymer dielectric materials make them promising candidates for next-generation memory devices. Ongoing research aim to address these hurdles and unlock the full potential of polymer dielectrics in advanced memory applications.

### 4.2. Challenges in the Area of Dielectric Polymers for Future FETs

Polymer dielectric materials have been used extensively in FET devices based on emerging semiconductors, as highlighted in this paper. Polymer materials have been designed to have high dielectric constant and reliable insulating performance to fabricate high-performance and low-power FETs. Some polymers can exhibit unique characteristics such as dopant properties, barrier properties, or ferroelectricity. Polymers have also been used as organic moieties in hybrid dielectrics, enhancing the FET performance, operational stability, or mechanical deformability. Nevertheless, there are still challenges and opportunities for polymer dielectric materials in FETs, as follows:

(1) The electrical properties should be further improved. Although a high dielectric constant and reliable insulating properties have been secured in some polymers, polymer dielectric materials are still considered inferior to inorganic counterparts in their electrical characteristics. Overcoming these limitations and developing polymer dielectric layers with high capacitance will need molecular-level material and structural design [5,36]. For example, increasing the dielectric constant by allowing polar functional groups to respond to the applied electric field may be necessary [114,117,121,126], and ferroelectric polymer can be a representative example of this design [171,182,188]. In addition, it is essential to densify the polymer matrix to minimize the leakage current path even at extremely thin thicknesses [115,124,127,128].
(2) Appropriate material design and process optimization are necessary. Polymer dielectric films are mostly fabricated through solution-based processing methods, which are advantageous in terms of process simplicity and potential printability [47,118,166]. On the other hand, solution processes can affect the electrical properties of polymer dielectric layers due to residual solvents or additives. Research has been performed on polymer dielectric materials based on chemical vapor deposition processes such as parylene [31,32,265]. Nevertheless, these processes require vacuum equipment and cost. Furthermore, reliable and uniform processability on a large scale should also be ensured to expand the practical application potential for polymer dielectric materials [9,50,164].

Despite these challenges, polymer dielectric materials are expected to be highly valuable because of their unique characteristics, such as tunability in chemistry and excellent mechanical properties, particularly in form factor-free, future electronics.

### 4.3. Challenges in the Area of Dielectric Polymers for Future TENGs

Polymer dielectric materials are used widely in TENGs to convert mechanical energy into electrical energy. These materials have been engineered to enhance dielectric properties and charge-trapping capabilities, which are essential for high-performance TENGs. Polymers, such as PVDF and its copolymers, have been popular because of their high dielectric constant and flexibility, allowing for efficient energy harvesting and mechanical adaptability. On the other hand, there are still challenges and opportunities for polymer dielectric materials in TENGs, as follows:

(1) Efficiency and output performance: Although some polymer dielectrics show promising triboelectric properties, achieving higher efficiency and output performance remains challenging. Enhancing the dielectric constant and surface charge density through material innovation and surface engineering is critical to improving the efficiency of TENGs. For example, integrating high-dielectric nanoparticles, such as BaTiO_3_ or TiO_2_, into the polymer matrix can significantly boost the output performance. In addition, producing nanocomposite structures that incorporate conductive fillers like Au nanoparticles can further enhance charge transport and storage capabilities, increasing the overall energy conversion efficiency of TENGs [266].
(2) Durability and wear resistance: TENGs are subject to repeated mechanical contact and friction, which can degrade polymer materials over time. Developing polymers with high mechanical durability and wear resistance is essential for ensuring long-term reliability and consistent performance [267]. Innovations can enhance durability, such as crosslinking polymer chains to produce a more robust network or adding wear-resistant additives. For example, incorporating silicone-based elastomers or other flexible yet tough materials can reduce surface wear and maintain the integrity of the triboelectric layers. Furthermore, applying protective coatings that resist abrasion and environmental degradation can help preserve the functional properties of the polymer dielectric materials in TENGs.
(3) Environmental stability: TENGs often operate under various environmental conditions, such as humidity, temperature fluctuations, and UV exposure. Ensuring that polymer dielectrics maintain their performance under these conditions requires the development of environmentally robust materials. Polymers with inherent environmental stability, such as fluorinated polymers or those that can be chemically modified to resist harsh conditions, will be crucial for the reliable operation of TENGs in diverse settings. For example, surface modification with hydrophobic coatings can prevent moisture absorption [268], while UV stabilizers protect the material from photodegradation. In addition, selecting polymers that have a broad operational temperature range can ensure consistent performance in both high- and low-temperature environments.

Despite these challenges, polymer dielectric materials are expected to be highly valuable for TENG applications because of their unique properties and potential for innovative designs. The ability to tailor polymer properties at the molecular level offers significant opportunities for optimizing TENG performance. Ongoing research and development are needed to overcome these hurdles and unlock the full potential of polymer dielectrics in next-generation energy-harvesting devices.

## 5. Conclusions and Outlook

Polymer dielectric materials have potential applications in memory devices, FETs, and TENGs. The integration and synergy among these devices can lead to innovative solutions and advancements in flexible and wearable electronics, self-powered systems, and sustainable technologies.

### 5.1. Interconnected Functionalities

(1) Memory devices and FETs: Polymer dielectric materials can be used to develop flexible, low-power memory devices that integrate seamlessly with FETs. The high dielectric constants and reliable insulating properties of polymers enable the development of memory–transistor hybrid systems that combine data storage with logic functions, such as logic-in-memory systems. This integration can result in compact, high-performance devices capable of performing complex computations while retaining data, all within a flexible form factor.
(2) TENGs and FETs: TENGs can be self-powered sources for FET-based electronic circuits and sensors. By converting mechanical energy from movements or environmental vibrations into electrical energy, TENGs can provide a sustainable power supply for FETs, eliminating the need for external batteries. This integration is beneficial for wearable sensors and remote monitoring devices where replacing or recharging batteries is impractical.

### 5.2. Specific Integration Strategies in Artificial Intelligence of Things (AIoT) Devices

Integrated systems that combine TENGs, FETs, and memory devices can play a crucial role in AIoT applications where continuous and reliable power supply is essential. These advanced systems can harness energy from environmental sources using TENGs, ensuring a sustainable power supply without external batteries. The harvested energy can power FETs for data processing and memory devices for data storage, enabling smart sensors and devices to function autonomously.

For example, smart infrastructure sensors can use TENGs to convert mechanical energy from vibrations or movements into electrical energy. This energy can power FETs that process real-time data and memory devices that store historical data. These AIoT devices can then use artificial intelligence algorithms to analyze the data, predict maintenance needs, and optimize performance. This integration allows for real-time decision making and adaptive responses to changing conditions, enhancing the efficiency and reliability of smart infrastructure.

In agricultural settings, AIoT devices can monitor soil conditions, weather patterns, and crop health. TENGs can harvest energy from wind, rain, or mechanical movements, powering the sensors and processors needed to collect and analyze data. Memory devices can store large datasets for longitudinal analysis, while AI algorithms can provide insights and recommendations for improving crop yields and resource management.

Wearable health monitors in the AIoT ecosystem can benefit from this integration by harvesting energy from the user’s movements. TENGs can generate power from everyday activities, enabling the continuous monitoring of vital signs without frequent recharging. The FETs process the health data in real time, and the memory devices store critical health information. AI algorithms can analyze data to detect anomalies, provide health recommendations, and alert users or healthcare providers to potential issues.

AIoT systems can achieve a high level of autonomy and intelligence by combining TENGs, FETs, and memory devices, operating efficiently in various environments and applications. This integration enhances the capabilities of IoT devices, making them more sustainable, intelligent, and responsive to real-world conditions.

## Figures and Tables

**Table 1:** Comparison of polymer dielectric-based memristor devices.

Active Materials (Method)	Resistance on/off Ratio	Switching Cycles	Retention Time (s)	Working Temperature (°C)	Air Stability (Days)	Biodegradable	[Ref.]
PVA (Spin-coating)	N/A	5 × 10^3^	10^4^	From 20 to 80	N/A	○	[71]
PPT^−^NMI^+^Br^− 1^ (Spin-coating)	N/A	10^2^	10^4^	Room temperature	N/A	○	[10]
MDMO-PPV (Spin-coating)	N/A	10^4^	10^4^	From −196 to 300	N/A	✕	[79]
PEI (Spin-coating)	~10^5^	10^3^	10^5^	From 25 to 150	N/A	✕	[76]
PTPA (Spin-coating)	~10^8^	<10	8 × 10^3^	From −243 to 117	N/A	✕	[77]
PEI-AgClO_4_ (Spin-coating)	~10^3^	5 × 10^2^	7 × 10^2^	Room temperature	30	✕	[83]
BPQDs@PDA-PVP ^3^ (Spin-coating)	N/A	2 × 10^2^	2 × 10^4^	Room temperature	90	✕	[84]
pV3D3 (iCVD)	~10^9^	10^5^	10^5^	From 27 to 85	N/A	✕	[51]
pV3D3 (iCVD)	~10^8^	10^5^	10^5^	From 27 to 87	27	✕	[85]
pEDGMA ^2^ (iCVD)	2.5 × 10^2^	5 × 10^2^	10^7^	Room temperature	N/A	✕	[86]

^1^ reactant of poly[thiophene-alt-4,4-bis(6-bromohexyl)-4H-cyclopenta(1,2-b:5,4-b′)dithiophene] (PTT-Br) with N-methylimidazole (NMI). ^2^ poly(ethylene glycol dimethacrylate) (pEDGMA). ^3^ blend of black phosphorus quantum dots (BPQDs) with polyvinylpyrrolidone-grafted polydopamine (PDA-PVP).

**Table 2:** Comparison of polymer dielectric-based charge trap devices.

Charge Trap Structure	Device Type	Current on/off Ratio	Memory Window (Operating Voltage)	Switching Cycles	Retention Time (s)	[Ref.]
pV3D3/AuNP/pC1D1	Floating gate	~10^6^	~5.7 V (±13 V)	1.5 × 10^3^	10^4^	[50]
pV3D3/Al/pEDGMA	Floating gate	10^6^	5.5 V (±10 V)	10^3^	3.2 × 10^8^	[55]
PMMA@F8BT/P(VDF-TrFE-CFE) ^2^	Floating gate	6.5 × 10^3^	9.3 V (±40 V)	10^2^	>10^4^	[93]
PFO	Polymer electrets	~10^7^	76 V (Light/−100 V)	N/A	~4 × 10^3^	[88]
PαMS ^1^	Polymer electrets	10^5^	23 V (±20 V)	5 × 10^3^	10^4^	[94]
pV3D3	Polymer electrets	>10^5^	5.3 V (±14 V)	50	>10^5^	[95]
PF-b-PDL	Polymer electrets	10^5^	102 V (±140 V)	N/A	10^4^	[21]
PF-b-Piso ^3^	Polymer electrets	10^6^	33 V (Light/−40 V)	10	10^4^	[91]
PVN PVN@2.5% PCBM PVN@30% PCBM	Polymer electrets	>10^4^ >10^4^ >10^4^	~12 V (±20 V) ~14 V (±20 V) ~15 V (±20 V)	N/A N/A N/A	10^3^ 10^3^ 10^3^	[22]
N2200@ PVN ^4^	Polymer electrets	10^5^	30 V (+30 V/−18 V)	10^3^	10^4^	[90]

^1^ poly(α-methylstyrene) (PαMS). ^2^ poly(9,9-dioctylflfluorene-co-benzothiadiazole) (F8BT) and poly(vinylidene flfluoride-triflfluoroethy-lene-chloroflfluoroethylene) (P(VDF-TrFE-CFE)). ^3^ poly [2,7-(9,9-dihexylfluorene)]-b- poly(pendent isoindigo) (PF-b-Piso). ^4^ blend of poly [96] (N2200) with poly(2-vinyl naphthalene) (PVN).
